# Supplementary material for: Knowledge of abortion legality among health facility staff in Ghana
Source: PLoS One. 2024 Aug 22;19(8):e0308371. doi: 10.1371/journal.pone.0308371 (PMC11340966; doi:10.1371/journal.pone.0308371)
Supplement: S1 File — (DOCX) [file pone.0308371.s001.docx]

STROBE Statement—checklist of items that should be included in reports of observational studies

|  | Item No. | Recommendation | Page  No. | Relevant text from manuscript |
| --- | --- | --- | --- | --- |
| **Title and abstract** | 1 | (*a*) Indicate the study’s design with a commonly used term in the title or the abstract | 2 | “Data for this paper are drawn from a nationally representative cross-sectional health facility survey conducted in 2018” |
|  |  | (*b*) Provide in the abstract an informative and balanced summary of what was done and what was found | 2 | **“Methods**  Data for this paper are drawn from a nationally representative cross-sectional health facility survey conducted in 2018; our analytic sample includes 340 facilities that provide induced abortion and/or postabortion care. The survey collected data on provision of abortion and postabortion care, as well as respondents’ knowledge of abortion legality and recommendations for reducing unsafe abortion. We used descriptive statistics to examine levels of knowledge and recommendations, and logistic regression to assess associations with individual and facility characteristics.  **Findings**  We find that comprehensive knowledge of the legal indications for abortion is low among health facility staff; just 6.3% of respondents identified all legal indications, and the majority (85%) underestimated the number of conditions under which abortion is legal. Knowledge was higher for more restrictive indications, such as a woman’s life being at risk, which was correctly identified by 71.5% of respondents, than more broadly interpretable indications, such as mental health, identified by 28.8% of respondents. Two-thirds of respondents recommended educating the public on the law.” |
| Introduction | | | |  |
| Background/rationale | 2 | Explain the scientific background and rationale for the investigation being reported | 3-5 | See background section |
| Objectives | 3 | State specific objectives, including any prespecified hypotheses | 5 | “We aimed to:   1. Explore levels of knowledge of abortion legality among staff in non-NGO health facilities offering post-abortion care (PAC) and/or induced abortion across Ghana; 2. Identify health facility staff recommendations for reducing unsafe abortion; 3. Identify respondent- and facility-level factors associated with knowledge of abortion legality and with recommendations for reducing unsafe abortion.” |
| Methods | | | |  |
| Study design | 4 | Present key elements of study design early in the paper | 6-10 | See methods section |
| Setting | 5 | Describe the setting, locations, and relevant dates, including periods of recruitment, exposure, follow-up, and data collection | 5-6 | See background and methods section |
| Participants | 6 | (*a*) *Cohort study*—Give the eligibility criteria, and the sources and methods of selection of participants. Describe methods of follow-up  *Case-control study*—Give the eligibility criteria, and the sources and methods of case ascertainment and control selection. Give the rationale for the choice of cases and controls  *Cross-sectional study*—Give the eligibility criteria, and the sources and methods of selection of participants | 6-7 | See methods section |
|  |  | (*b*) *Cohort study*—For matched studies, give matching criteria and number of exposed and unexposed  *Case-control study*—For matched studies, give matching criteria and the number of controls per case | n/a |  |
| Variables | 7 | Clearly define all outcomes, exposures, predictors, potential confounders, and effect modifiers. Give diagnostic criteria, if applicable | 7-10 | Detailed description of dependent and independent variables included in methods section |
| Data sources/ measurement | 8* | For each variable of interest, give sources of data and details of methods of assessment (measurement). Describe comparability of assessment methods if there is more than one group | 7-10 |  |
| Bias | 9 | Describe any efforts to address potential sources of bias | 9-10 | “We considered a range of respondent- and facility-level characteristics as independent variables. Respondent-level covariates included age (19-29/30-39/40+), gender (male/female), position (physician, midlevel provider, other) and years in current occupation (0-5 years/6+ years). Facility-level covariates included facility level (primary vs. secondary/tertiary), sector (public/private), location (urban/rural), and provision of post-abortion care versus provision of post-abortion care and/or induced abortion.” |
| Study size | 10 | Explain how the study size was arrived at | 6-7 | “Data for this analysis come from a nationally representative health facility survey conducted from June through September 2018. The Central Health Information Management System of the Ghana Health Service was used to obtain a list of health facilities that reported data through the District Health Information Management system in 2017. All facility types that could potentially provide abortion and/or post-abortion care, except for community-based health planning and services facilities and non-governmental organizations (NGOs), were retained. The sample frame included 2,758 facilities; complete details on sampling are provided elsewhere (Polis et al. 2020). All teaching and regional hospitals were sampled while for lower-level facilities, a two-stage stratified cluster sampling design, by facility level was used to generate a nationally representative sample. A total of 539 facilities completed the survey, an 88.7% response rate (Polis et al., 2020). Our analytic sample included the 340 facilities who reported providing postabortion care, induced abortion care, or both, since only respondents from these facilities were administered the complete survey, including questions on the conditions under which abortion is legal.” |

Continued on next page

| Quantitative variables | 11 | Explain how quantitative variables were handled in the analyses. If applicable, describe which groupings were chosen and why | 7-10 | Detailed description provided in methods section. |
| --- | --- | --- | --- | --- |
| Statistical methods | 12 | (*a*) Describe all statistical methods, including those used to control for confounding | 10 | See analysis section. |
|  |  | (*b*) Describe any methods used to examine subgroups and interactions |  |  |
|  |  | (*c*) Explain how missing data were addressed |  |  |
|  |  | (*d*) *Cohort study*—If applicable, explain how loss to follow-up was addressed  *Case-control study*—If applicable, explain how matching of cases and controls was addressed  *Cross-sectional study*—If applicable, describe analytical methods taking account of sampling strategy |  |  |
|  |  | (*e*) Describe any sensitivity analyses |  |  |
| Results | | | | |
| Participants | 13* | (a) Report numbers of individuals at each stage of study—eg numbers potentially eligible, examined for eligibility, confirmed eligible, included in the study, completing follow-up, and analysed | 6-7 | “Data for this analysis come from a nationally representative health facility survey conducted from June through September 2018. The Central Health Information Management System of the Ghana Health Service was used to obtain a list of health facilities that reported data through the District Health Information Management system in 2017. All facility types that could potentially provide abortion and/or post-abortion care, except for community-based health planning and services facilities and non-governmental organizations (NGOs), were retained. The sample frame included 2,758 facilities; complete details on sampling are provided elsewhere (Polis et al. 2020). All teaching and regional hospitals were sampled while for lower-level facilities, a two-stage stratified cluster sampling design, by facility level was used to generate a nationally representative sample. A total of 539 facilities completed the survey, an 88.7% response rate (Polis et al., 2020). Our analytic sample included the 340 facilities who reported providing postabortion care, induced abortion care, or both, since only respondents from these facilities were administered the complete survey, including questions on the conditions under which abortion is legal.” |
|  |  | (b) Give reasons for non-participation at each stage | 6-7 | See above. |
|  |  | (c) Consider use of a flow diagram |  |  |
| Descriptive data | 14* | (a) Give characteristics of study participants (eg demographic, clinical, social) and information on exposures and potential confounders | 11 | “The characteristics of the 340 respondents, each representing a facility, are presented in Table 1. Nearly half (48.4%) of respondents were over the age of 40, and 66.3% were female. Most respondents (75.8%) were midlevel providers, while 19.3% were physicians. More than half (58.5%) had been in their occupation for more than six years. Half of respondents (55.9%) worked in primary-level facilities. There was a nearly even divide between public and private sector facilities (48.3% and 51.7% respectively), and slightly more facilities were in urban areas (52.4%). All facilities in our sample provided postabortion and/or induced abortion care: 64.7% provided PAC only, 34.6% provided both PAC and abortion, and 0.7% provided only induced abortion.” |
|  |  | (b) Indicate number of participants with missing data for each variable of interest |  | Described in methods and noted where relevant in results. |
|  |  | (c) *Cohort study*—Summarise follow-up time (eg, average and total amount) | N/A |  |
| Outcome data | 15* | *Cohort study*—Report numbers of outcome events or summary measures over time |  |  |
|  |  | *Case-control study—*Report numbers in each exposure category, or summary measures of exposure |  |  |
|  |  | *Cross-sectional study—*Report numbers of outcome events or summary measures | 12 | See results section for detailed reporting of outcomes and summary measures. |
| Main results | 16 | (*a*) Give unadjusted estimates and, if applicable, confounder-adjusted estimates and their precision (eg, 95% confidence interval). Make clear which confounders were adjusted for and why they were included | 15-19 | See Tables 3, 4, and 5 for presentation for unadjusted and adjusted estimates with 95% confidence intervals. All confounders are outlined in each table. |
|  |  | (*b*) Report category boundaries when continuous variables were categorized | 9-10 | Categorization of continuous variables is described in the methods section. |
|  |  | (*c*) If relevant, consider translating estimates of relative risk into absolute risk for a meaningful time period |  |  |

Continued on next page

| Other analyses | 17 | Report other analyses done—eg analyses of subgroups and interactions, and sensitivity analyses | 10 | Full description of analysis procedures provided on page 10. |
| --- | --- | --- | --- | --- |
| Discussion | | | | |
| Key results | 18 | Summarise key results with reference to study objectives | 20 | “We found that complete and accurate knowledge of the legal conditions permitting abortion in Ghana was low among staff in health facilities offering abortion or post-abortion care, with considerable variation across conditions: while most respondents knew some of the circumstances under which abortion is legal, few had comprehensive knowledge of the legal conditions, and 85% underestimated the number of conditions for which abortion is legal. Knowledge was considerably higher for more restrictive indications (e.g., if a woman’s life is at risk) while other, more broadly interpretable indications (e.g., mental health) were less known. Those working in facilities that provided induced abortion care in addition to PAC often had better knowledge of the legal indications for abortion than those working in facilities providing only PAC, and were less likely to underestimate abortion legality; this could reflect exposure to a wider range of clients seeking abortion care for different legal indications. Those working in the public sector and with more recent training often had better knowledge of abortion legality as well.” |
| Limitations | 19 | Discuss limitations of the study, taking into account sources of potential bias or imprecision. Discuss both direction and magnitude of any potential bias | 23 | “…our study has limitations. We were unable to assess knowledge of legality among staff from facilities that do not provide abortion or post-abortion care, who would likely have poorer knowledge of the law. Our sample excludes NGO and CHPS facilities, which comprise 24.9% of abortion-providing facilities in Ghana, and this may bias our estimates if knowledge among this population differs substantially. Our measure of knowledge of the law did not have response options read aloud, and it is possible that with probing on various legal indications, respondents’ knowledge of the law would have appeared greater. We also decided to group some legal indications in our analysis (e.g., threat to life and health) to simplify the presentation of our results rather than present more precise regression results for each legal indication, which may impede the comparison of differences between the legal indications.” |
| Interpretation | 20 | Give a cautious overall interpretation of results considering objectives, limitations, multiplicity of analyses, results from similar studies, and other relevant evidence | 23-24 | “While many health facility staff in Ghana knew some legal indications for abortion, complete knowledge of the abortion law was low, and most respondents underestimated the legality of abortion. Knowledge of indications with broader interpretability, i.e., mental health, was particularly low. Knowledge of the law among health providers and other facility staff is important for ensuring abortion care is made available to the fullest extent of the law in Ghana. Efforts are needed to improve knowledge of the law among providers and facility staff, particularly for health indications and their potentially broad interpretability.” |
| Generalisability | 21 | Discuss the generalisability (external validity) of the study results | 23 | “Our study provides the first nationally representative estimates of knowledge of abortion legality among a range of providers and staff in health facilities offering abortion or post-abortion care in Ghana, excluding NGO and community-based health planning and services (CHPS) facility personnel. We were able to explore physicians, midlevel providers, and administrators’ knowledge of each condition of the abortion law which provides a more complete understanding of existing gaps in knowledge.” |
| Other information | |  | | |
| Funding | 22 | Give the source of funding and the role of the funders for the present study and, if applicable, for the original study on which the present article is based |  | Included in financial disclosure statement as supplementary file. |

*Give information separately for cases and controls in case-control studies and, if applicable, for exposed and unexposed groups in cohort and cross-sectional studies.

**Note:** An Explanation and Elaboration article discusses each checklist item and gives methodological background and published examples of transparent reporting. The STROBE checklist is best used in conjunction with this article (freely available on the Web sites of PLoS Medicine at http://www.plosmedicine.org/, Annals of Internal Medicine at http://www.annals.org/, and Epidemiology at http://www.epidem.com/). Information on the STROBE Initiative is available at www.strobe-statement.org.
