# Supplementary material for: Knowledge of abortion legality among health facility staff in Ghana
Source: PLoS One. 2024 Aug 22;19(8):e0308371. doi: 10.1371/journal.pone.0308371 (PMC11340966; doi:10.1371/journal.pone.0308371)
Supplement: Attachment — Submitted filename: PLOS_Response_Letter_06.04.2024.docx [file pone.0308371.s004.docx]

PONE-D-23-17111

Knowledge of abortion legality among health facility staff in Ghana

PLOS ONE

June 4, 2024

Dear PLOS ONE Editors,

Thank you for the opportunity to revise and resubmit our manuscript, *Knowledge of abortion legality among health facility staff in Ghana*. Below we have responded to the recommended revisions. We look forward to hearing back with a final decision on the manuscript.

Best wishes,

Grace Sheehy

**Editor’s feedback:**

Upon reevaluation, I find your response to be thorough. However, I believe the introduction section could benefit from further enhancement, incorporating insights from the following papers:

1. Morhee, R. A. S., & Morhee, E. S. K. (2006). Overview of the law and availability of abortion services in Ghana. Ghana medical journal, 40(3).
2. Aniteye, P., & Mayhew, S. H. (2013). Shaping legal abortion provision in Ghana: using policy theory to understand provider-related obstacles to policy implementation. Health Research Policy and Systems, 11, 1-14.
3. Abubakari, A., Amankwa, A. M., Owusu, D., Fosuah, F., & Anane, J. Utilization of Comprehensive Abortion Care (CAC) and associated factors among young people (10-24 years): a cross-sectional study in the Tamale Metropolis. Northern Region of Ghana.
4. Abdul-Wahab, I., MubarickA., Nimota Nukpezah, R., & Kojo Dzantor, E. (2021). Adolescents' sexual and reproductive health: A survey of knowledge, attitudes, and practices in the Tamale Metropolis, Ghana.

I kindly request that you refine the introduction for clarity and conciseness.

**Authors’ response to editor:**

Thank you for your suggestions for strengthening the introduction section. We have incorporated three of the proposed references into this section (Morhee & Morhee 2006; Aniteye & Mayhew 2013; Abubakari et al. 2023) and have made some revisions for clarity and conciseness throughout the introduction.
